# Evaluation of Carer Contact Practices for Junction 17 CAMHS Inpatient Unit

**DOI:** 10.1192/bjo.2025.10439

**Published:** 2025-06-20

**Authors:** Alaaelrahman Shahib, Ashley Cooper, Rebecca Dugard

**Affiliations:** Mersey and West Lancashire NHS Trust, Greater Manchester, United Kingdom

## Abstract

**Aims:** This audit aimed to assess and improve the consistency and quality of carer contact within the CAMHS inpatient ward at Junction 17. The objectives were to evaluate current communication practices against Trust policies, ensure effective documentation of carer interactions, and implement strategies to improve adherence to guidelines.

**Methods:** A retrospective audit was conducted on 12 CAMHS inpatients admitted between April 1, 2024, and June 1, 2024. Data was extracted from patient records on the PARIS system and ePMA, focusing on key carer contact indicators:

Assignment of a staff member for carer communication.

Documentation of consent for carer contact.

Carer contact within 72 hours of admission.

Communication regarding medication/management changes.

Carer contact following incidents, home leaves, and ward rounds.

Adherence rates were calculated, and qualitative analysis was conducted to assess documentation clarity and consistency.

**Results:** Assignment of Staff Member: Only 58% (7/12) of patients had a clearly assigned staff member responsible for carer contact.

Consent Documentation: While 100% of patients had verbal consent obtained, only 8% (1/12) had clear documentation in the PARIS system.

Carer Contact Compliance:

Within 72 hours of admission: 100% compliance.

Post-medication/management changes: 2/3 (66%) compliance.

Post-incident contact: 73% (132/180 incidents) compliance.

Post-home leave: 87% compliance.

Post-ward round: Only 47% (51/108) compliance.

Common issues included inconsistent documentation, difficulty in retrieving records, and lack of designated time for carer communication.

**Conclusion:** While initial carer contact post-admission and post-home leave showed high compliance, significant gaps were identified in staff assignment, documentation of consent, and post-ward round communication. Key recommendations include:

Assigning primary and secondary staff members as key family links.

Standardizing documentation in the PARIS system for easier retrieval.

Allocating protected time for carer contact, particularly post-ward rounds.

Integrating carer communication into the CAMHS staff induction programme.

A re-audit is scheduled in three months to assess the impact of these interventions. If successful, the proposed guidelines will be considered for broader implementation at a national level.

